# Editorial: Horticultural genetic resources

**DOI:** 10.3389/fpls.2023.1264597

**Published:** 2023-08-21

**Authors:** Mostafa Farajpour, Mohammad M. Arab, Ramesh Katam, Mohammad Sadat-Hosseini

**Affiliations:** ^1^ Crop and Horticultural Science Research Department, Mazandaran Agricultural and Natural Resources Research and Education Center, Agricultural Research, Education, and Extension Organization (AREEO), Sari, Iran; ^2^ Department of Horticulture, College of Aburaihan, University of Tehran, Tehran, Iran; ^3^ Department of Biological Sciences, Florida A&M University, Tallahassee, FL, United States; ^4^ Department of Horticulture, Faculty of Agriculture, University of Jiroft, Jiroft, Iran

**Keywords:** genetic resource, climate change, environmental stress, biotechnology, horticultural crop

Horticultural crops play a vital role in human diets, providing essential nutrients and contributing to food security, economic stability, and cultural value. However, horticultural production is facing numerous challenges due to climate change and other environmental stresses in the past few decades. These challenges include increased temperatures, reduced precipitation, soil salinity, extreme weather events, and emerging pests and diseases. Therefore, genetic resources are vital to developing more resilient horticultural varieties that adapt to changing environmental conditions.

Integrating new OMICS and phenotyping approaches in horticultural genetic resources conservation and utilization has revolutionized our understanding of plant diversity and breeding. Genomic approaches enable rapid analysis of DNA sequences, identifying genetic variations, and exploring gene functions and metabolic pathways. Phenomics approaches measure plant traits at a large scale, providing valuable data on genotype-environment interactions. These tools help breeders identify superior horticultural varieties with desired traits, such as improved yield and resilience. In addition, genome editing technologies like CRISPR-Cas9 offer precise modifications to the plant genome, accelerating breeding and introducing beneficial traits.

Recent biotechnological advancements, such as next-generation sequencing and genomics-based approaches, have enabled the selection of potential parents from germplasm collections. These methods also include Omics approaches, like transcriptomics, proteomics, and metabolomics, to create new cultivars with higher adaptability to environmental conditions.

Breeding resilient cultivars depends on genetic resources and cutting-edge technologies. Next-generation sequencing allows for cost-effective analysis of DNA sequences to identify significant variations and genes linked to desirable traits. High-throughput phenotyping platforms provide a comprehensive understanding of phenotypic variations and their interactions with the environment, improving breeders’ knowledge of plant morphology, physiology, and biochemical composition. Advancements in genotyping platforms facilitate the identification of genetic variations linked to important traits, supporting genomics-based approaches like GWAS, MAS, and GS. Multi-omics approaches, such as transcriptomics, metabolomics, and proteomics, expand our understanding of complex biological processes underlying horticultural traits. Integrating horticultural genetic resources with next-generation genomics and phenomics technologies enables breeders to develop new cultivars with improved adaptability, productivity, and quality, ensuring resilience in the face of climate change.

Genetic engineering techniques have led to the development of GMOs by transferring desirable genes between plant species to improve traits like pest resistance, disease resistance, herbicide resistance, and nutritional quality. However, the use of GMOs is controversial due to safety and environmental concerns. With CRISPR-Cas9 gene editing, precise modifications can be made to the plant genome to introduce beneficial traits and remove undesirable ones.

Recent research and developments in using genetic resources for horticultural crops have focused on various aspects of breeding for resilience, conservation of genetic resources, and molecular tools for selection.

For example, Ahmad et al. demonstrated a considerable diversity in phenological and biochemical characteristics of date palm cultivars and a correlation among several elements of the studied germplasm, which can be exploited in future breeding programs.

Furthermore, studies have focused on the identifying genes associated with desirable traits, such as disease resistance and abiotic stress tolerance. For example, the identification of the NAC25 gene in coffee has revealed its independent evolution in coffee species, providing valuable insights into the genetic diversity of this crop (Huded et al.). In addition, the identification of cold-tolerant Iranian olive varieties has provided a potential solution for establishing olive groves under cold climate conditions (Karamatlou et al.). In another report, for genetic polymorphisms evaluation, researchers applied the K-seq protocol in both ornamental species, including *Ranunculus asiaticus* L. and *Anemone coronaria* L. Finally, 11,542 SNPs were chosen for the identification of genetic variability of eighteen commercial varieties of *R. asiaticus*, while 1,752 SNPs for identification genetic variability in six cultivars of *A. coronaria* (Martina et al.).

Epigenetic modifications are changes in gene expression that are not caused by changes in the DNA sequence but are induced by environmental factors, contributing to the development of desirable traits. Biotechnological advancements, such as next-generation sequencing and genomics-based approaches, have enabled the selection of potential parents from germplasm collections. High-throughput genotyping platforms and mapping techniques facilitate the identification of genes associated with desirable traits. However, challenges to effective utilization of genetic resources include limited access for small-scale farmers and breeders in developing countries and commercialization leading to monopolization by a few companies, limiting their availability to others.

Therefore, efforts are being made to address these challenges and promote the effective utilization of genetic resources. These efforts include the development of international agreements, such as the International Treaty on Plant Genetic Resources for Food and Agriculture, which aims to ensure the conservation and sustainable use of plant genetic resources. In addition, there are initiatives to promote the sharing of genetic resources among countries and institutions, such as the Global Crop Diversity Trust and the CGIAR System Organization.

## Author contributions

MS-H: Writing – original draft, Writing – review & editing, MA: Writing – original draft, Writing – review & editing, RK: Writing – original draft, Writing – review & editing. MF: Conceptualization, Writing – original draft, Writing – review & editing.

